# mTOR Inhibitor Use Is Associated With a Favorable Outcome of COVID-19 in Patients of Kidney Transplant: Results of a Retrospective Study

**DOI:** 10.3389/fmed.2022.852973

**Published:** 2022-06-21

**Authors:** Biagio Pinchera, Lorenzo Spirito, Antonio Riccardo Buonomo, Maria Foggia, Rosa Carrano, Fabrizio Salemi, Elisa Schettino, Fortuna Papa, Roberto La Rocca, Felice Crocetto, Luigi Napolitano, Riccardo Villari, Ivan Gentile

**Affiliations:** ^1^Section of Infectious Diseases, Department of Clinical Medicine and Surgery, University of Naples Federico II, Naples, Italy; ^2^Section of Urology, Department of Neurosciences, Reproductive and Odontostomatological Sciences, University of Naples Federico II, Naples, Italy; ^3^Section of Nephrology, Department of Public Health, University of Naples Federico II, Naples, Italy

**Keywords:** kidney transplant, SARS-CoV-2, COVID-19, mTOR inhibitors, immunosuppressive therapy

## Abstract

**Introduction:**

In solid organ transplant recipients, COVID-19 is associated with a poor prognosis because of immunosuppression. Some studies suggest a potential therapeutic role of mammalian Target of Rapamycin (mTOR) inhibitors in SARS-CoV-2 infection. This study aimed to assess the impact of mTOR employment on the evolution and outcome of SARS-CoV-2 infection in solid organ transplant recipients.

**Methods:**

We enrolled kidney transplant patients attending the Azienda Ospedaliera Universitaria Federico II in Naples and followed up on these patients from March 2020 to June 2021. We evaluated the risk of acquiring the SARS-CoV-2 infection, the clinical presentation of the disease, and its outcome together with the type of immunosuppressive therapy. Finally, we assessed the impact of mTOR inhibitors on relevant clinical metrics of SARS-CoV-2 infection.

**Results:**

We enrolled 371 patients, of whom 56 (15.1%) contracted SARS-CoV-2 infection during the period of the study. There were no differences observed among the different immunosuppressive therapies concerning the risk of acquiring SARS-CoV-2 infection. In contrast, the type of immunosuppressive therapy had a significant impact on the outcome of the disease. In detail, patients who received mTOR inhibitors, as part of their immunosuppressive therapy, compared to other regimens had a lower chance of developing a moderate or severe form of the disease (OR = 0.8, 95, CI: (0.21–0.92), *P* = 0.041).

**Conclusion:**

In kidney transplant patients, the use of mTOR inhibitors as part of an immunosuppressive regimen is associated with a better prognosis in the case of COVID-19.

## Introduction

Immunosuppressive therapy is a crucial aspect in a solid organ transplant patient. It is the mainstay of the prevention of rejection of the allograft, but at the same time, it contributes to determining the patient's susceptibility to several infections (1–3). Different immunosuppressive drugs, such as calcineurin inhibitors (tacrolimus and cyclosporine), corticosteroids, antimetabolite agents (mycophenolate and azathioprine), and the mammalian target Of rapamycin (mTOR) inhibitors (everolimus and sirolimus), are used to prevent rejection (4, 5). In particular, mTOR is a crucial pathway in many physiological processes (such as cell cycle progression, transcription, translation, differentiation, apoptosis, motility, and cell metabolism) and, therefore, plays a central role in the regulation of cell growth and proliferation, at the translational level, and in cell cycle progression. Moreover, as mTOR also modulates protein synthesis at ribosomal and transfer RNA transcription levels, it also plays a fundamental role in viral translation (6). It is already known that several viruses, such as adenovirus, cytomegalovirus, herpes simplex virus, and Middle East respiratory syndrome coronavirus (MERS – CoV), use the mTOR pathway to replicate (7, 8). The mTOR pathway is also involved in the life cycle of SARS-CoV-2 infection (9). The antiviral properties of mTOR have been known and ascribed to a variety of mechanisms (10). This aspect needs to be considered in relation to the pandemic impact (2–4). There are scarce data on the possible role of mTOR inhibitors vs. SARS-CoV-2 and their potential impact on the evolution of the disease; however, some studies support the potential therapeutic role of these drugs (11). Some reviews suggest the therapeutic potential of mTOR inhibitors, such as rapamycin, against COVID-19 both *in vitro* and *in vivo* (12–14).

For these reasons, blocking the mTOR signaling pathway could be a strategy to treat SARS-CoV-2 infection and its evolution. This study aimed to describe and assess the impact of the mTOR inhibitor therapy on the evolution and outcome of SARS-CoV-2 infection in solid organ transplant recipients followed in our center.

## Materials and Methods

We conducted an observational retrospective cohort study. We enrolled patients with kidney transplants attending the Azienda Ospedaliera Universitaria Federico II in Naples from March 2020 to June 2021. Diagnosis of SARS-CoV-2 infection was obtained by positivity to the rhino-oropharyngeal swab for SARS-CoV-2 RNA research by reverse transcription-polymerase chain reaction (RT-PCR). For patients with COVID-19, we used the Henry Ford Hospital (HFH). COVID-19 severity scoring system to distinguish the disease's mild, moderate, and severe forms (15). In particular, the mild disease was defined as patients who had normal chest radiography and SpO^2^ of ≥94% without the need for supplemental oxygen. Patients with moderate disease were those who had abnormal chest radiography, SpO^2^ of <94%, and were in need of 1 and 5 L/min supplemental O^2^. Patients with severe disease were defined by abnormal chest radiography, SpO2 of <94%, and requiring ≥6 L/min of O^2^ (16). For each patient, we evaluated the epidemiological characteristics, the laboratory data, the data of radiological instrumental investigations, clinical characteristics, the time elapsed since the transplant, the type of immunosuppressive treatment, and their changes during SARS-CoV-2 infection, the need for treatment and the type of treatment for SARS infection -CoV-2, and the outcome. In particular, we evaluated the potential relationship between the use of mTOR vs. other immunosuppressive regimens and severity or clinical outcome.

Data were reported as the median and interquartile range (IQR) given their non-parametric distribution. For comparisons between continuous variables, the Mann-Whitney U test was performed. We used the Chi-square test to test if two categorical variables are associated. Co-variates significantly associated with death in the univariate analysis were also analyzed in a multivariate model. The *P*-value for statistical significance was set at <0.05 for all the tests.

With respect to the ethical issues, the study was conducted in compliance with the Declaration of Helsinki and the principles of good clinical practice. The authors confirm adherence to the ethical policies of the journal, as noted on the journal's author guidelines page.

## Results

We enrolled 371 patients with kidney transplant (229 men, 61.8%) with a median age of 49 (18–86) years and a mean age of 51.4 years. Of these, 56 (15.1%) became infected with SARS-CoV-2 during the period of the study. Of these 56 patients with SARS-CoV-2 infection, 30 (53.6%) showed symptoms of the disease (COVID-19) and 26 had an asymptomatic infection (Table 1). Of the 30 patients with COVID-19 symptoms, 15 (50%) had a mild form of the disease, 7 (24%) had a moderate form of the disease, and 8 (26%) had severe form of the disease. Hospitalization was necessary for 12 (21.4%) patients, eight with the severe form of the disease and four with the moderate one. Of the 12 patients admitted, five required oxygen supplementations, five required non-invasive/high flow ventilation, and two required invasive ventilation (Table 1). Of the enrolled patients, only 12 patients performed high-resolution lung computed tomography (HRCT); in particular, only hospitalized patients performed HRCT. The severity score index, as proposed by Chung et al. (17) was used for the analysis of each individual HRCT. The 12 patients had a median severity score index equal to 13/20 as proposed by Chung et al. (17). Of the 12 patients, only one was taking mTOR inhibitors, particularly sirolimus, and had a severity score index equal to 10/20, as proposed by Chung et al. (17). Distinguishing the severity score index, proposed by Chung et al. (17) between the group of mTOR inhibitors and the group without mTOR inhibitors (10/20 vs. 13/20), a severity score index, proposed by Chung et al. (17) was higher among patients without mTOR inhibitors.

**Table 1:** Anagraphical and clinical features of patients with kidney transplant with SARS-CoV-2 infection.

**Age (median, IQR)**	**50 (18–71)**
**Gender**	
Men	43 (76.7%)
Women	13 (23.3%)
**Asymptomatic**	**26 (46.4%)**
Men	22 (84.6%)
Women	4 (15.4%)
**COVID-19**	**30 (53.6%)**
Men	21 (70%)
Women	9 (30%)
**Comorbidities:**	
Hypertension	53 (94.6%)
Dyslipidemia	31 (55.3%)
Diabetes	10 (17.9%)
Anemia	14 (25%)
Ischemic heart disease	1 (1.78%)
**Therapy for COVID-19:**	
Modifications of immunosuppressive therapy	30 (100%)
Steroid therapy	22 (73.3%)
Low molecular weight heparin	18 (60%)
Remdesivir	4 (13.4%)

All 371 patients underwent immunosuppressive therapy at the time of enrollment. In particular, 220 underwent triple immunosuppressive therapy, 142 dual therapy, and nine single immunosuppressant (Tables 2–4).

**Table 2:** Immunosuppressive therapy for each patient.

**Immunosuppressive therapy**				
	**No SARS-CoV-2 infection**	**SARS-CoV-2 infection**		
		**Asymptomatic**	**COVID-19**	
			**Mild**	**Moderate/Severe**
Tacrolimus + mycophenolate + methylprednisolone	49	6	1	2
Tacrolimus + mycophenolate + prednisone	72	4		2
Tacrolimus + everolimus + methylprednisolone	10	1	1	
Tacrolimus + everolimus + prednisone	4	1		
Tacrolimus + sirolimus + methylprednisolone	1			
Tacrolimus + everolimus + mycophenolate + methylprednisolone	1			
Tacrolimus + everolimus	2	1	1	
Tacrolimus + sirolimus	1			
Cyclosporine + everolimus + methylprednisolone	6			
Cyclosporine + sirolimus + methylprednisolone	1			
Cyclosporine + everolimus + mycophenolate	1			
Cyclosporine + everolimus + prednisone	5		1	
Cyclosporine + everolimus	4		2	
cyclosporine + sirolimus		1		1
Sirolimus + methylprednisolone	10		1	
Everolimus + mycophenolate	1			
Sirolimus + mycophenolate + prednisone	2			
Everolimus + prednisone	4			
Sirolimus + prednisone	1			
Sirolimus	1			
Other immunosuppressive therapies without mTOR inhibitors	137	12	8	10
	315	26	15	15

**Table 3:** Single vs. double vs. triple immunosuppressive therapy.

**Immunosuppressive therapy**					
		**No SARS-CoV-2 infection**	**SARS-CoV-2 infection**		
			**Asymptomatic**	**COVID-19**	
				**Mild**	**Moderate/Severe**
Single	9 (2%)	9 (2.4%)	0	0	0
Double	142 (38%)	112 (30%)	11 (3%)	12 (3.2%)	7 (1.8%)
Triple	220 (60%)	194 (52.6%)	15 (4%)	3 (0.8%)	8 (2.2%)

**Table 4:** Single vs. dual vs. triple immunosuppressive therapy in patients with SARS-CoV-2 infection.

**Immunosuppressive therapy**				
		**SARS-CoV-2 infection**		
		**Asymptomatic**	**COVID-19**	
			**Mild**	**Moderate/Severe**
Double	30 (53.6%)	11 (19.6%)	12 (21.4%)	7 (12.5%)
Triple	26 (46.4%)	15 (26.8%)	3 (5.3%)	8 (14.4%)

Data concerning the different immunosuppressive regimens also in relation to clinical presentation and outcome are given in Tables 3, **6**.

In relation to the different immunosuppressive therapies, 66 patients (17.8%) assumed the immunosuppressive therapy with mTOR inhibitors, 48 with everolimus, and 18 with sirolimus. Of these, 11 (16.6%) (eight treated with everolimus and three with sirolimus) acquired SARS-CoV-2 infection (*OR for SARS-CoV-2 infection acquired vs. no SARS-CoV-2 infection acquired: 1.1, 95, CI: (0.25**–**2.8) mTOR inhibitors recipients vs. other regimens), p = 0.210*). Of the 11 patients infected, 7 (63.6%) had COVID-19; in particular, six had a mild form of the disease, while 1 had a moderate form of the disease (*OR for moderate/severe form vs. mild:0.8, 95, CI: (0.21**–**0.92) mTOR inhibitors recipients vs. other regimens; p = 0.041*) (Tables 2, 5, 6). No patient treated with mTOR inhibitors presented a severe form of the disease.

**Table 5:** Immunosuppressive therapy evaluation for single immunosuppressant.

**Immunosuppressive therapy**					
		**No SARS-CoV-2 infection**	**SARS-CoV-2 infection**		
			**Asymptomatic**	**COVID-19**	
				**Mild**	**Moderate/Severe**
Tacrolimus	247	212	19	9	7
Cyclosporine	92	73	7	4	8
Mycophenolate	209	181	16	3	9
Azathioprine	4	3	0	0	1
Everolimus	48	40	3	5	0
Sirolimus	18	15	1	1	1
Methylprednisolone	182	165	11	9	7
Prednisone	136	120	9	2	5

**Table 6:** Immunosuppressive therapy evaluation: mTOR inhibitors vs. other types of immunosuppressive therapies.

**Immunosuppressive therapy**					
		**No SARS-CoV-2 infection**	**SARS-CoV-2 infection**		
			**Asymptomatic**	**COVID-19**	
				**Mild**	Moderate/Severe^*^
mTOR inhibitors	66 (17.7%)	55 (83%)	4 (6%)	6 (9%)	1 (2%)
Other types of immunosuppressive therapies	305 (82.3%)	260 (85%)	22 (7%)	9 (3%)	14 (5%)

* *Moderate/severe vs. asymptomatic/mild P = 0.041*.

No significant differences were observed between those patients who received a triple vs. a mono/double immunosuppressive regimen in the risk of acquiring the infection (*OR = 1.1, 95, CI: (0.60*–*2.5), p = 0.270*) (Tables 3, 4).

All patients with symptoms underwent modifications of the immunosuppressive therapy. Regarding the therapeutic management of the infection, the first step was the reduction of immunosuppressive therapy, providing, in the first instance, the reduction or suspension of antimetabolites. In the case of severe forms of the disease, all immunosuppressive therapy was suspended, except for the steroid therapy, which was increased (*OR for modification/suspension of immunosuppressive therapy vs. non-modification of immunosuppressive therapy in SOT with a moderate-severe form of COVID-19:0.7, 95, CI: (0.44*–*0.85), p = 0.048*) (18–21). Only one patient experienced acute organ rejection, and two patients died.

We conducted a multivariate analysis of the possible variables that could impact the evolution of the COVID-19 disease, regardless of the presence or absence of mTOR inhibitors. We considered diabetes, BMI, duration of immunosuppressive treatment, duration of renal disease, and concomitant heart disease as variables. The multivariate analysis highlighted the values of diabetes [OR = 0.9, 95, CI: (0.85**–**1.4), *P* = 0.130], BMI [OR = 1.1, 95, CI: (0.92**–**1.3), *P* = 0.145], duration of immunosuppressive treatment [OR = 1.2, 95, CI: (0.72-1.8), *P* = 0.350], duration of renal disease [OR = 1.1, 95, CI: (0.52**–**2.1), *P* = 0.420], and concomitant heart disease [OR = 0.96, 95, CI: (0.88**–**1.7), *P* = 0.290].

## Discussion

Our study first shows that the use of mTOR inhibitors when compared with other immunosuppressive regiments was associated with a more favorable outcome of COVID-19 in a cohort of patients. Moreover, none of the patients undergoing immunosuppressive therapy with mTOR inhibitors (everolimus and sirolimus) presented a severe form of the disease.

In contrast, neither the number of immunosuppressive drugs nor their type was associated with the risk of acquiring the infection.

We underline that our data may add knowledge to the management of SARS-CoV-2 infection in patients who underwent solid organ transplant and, in particular, to the management of immunosuppressive therapy during this infection. Moreover, from our study, the role of mTOR inhibitors in COVID-19 treatment could be hypothesized even in a non-transplant setting. However, this hypothesis needs to be deepened and demonstrated with further studies with a different design (i.e., randomized controlled trial).

How can we explain these results? There are at least two possible explanations: an antiviral effect of mTOR inhibitors or an immunomodulant action. With respect to the first hypothesis, we underline that a potential positive impact of mTOR inhibitors in the course of several viral infections is already known in the literature (22, 23). However, to our best knowledge, our study is the first one to show a positive impact of mTOR inhibitors in the course of SARS-CoV-2 infection on the evolution of the disease. The results might be due to the wellknown immunomodulatory effect of these drugs that could reduce the cytokine storm typical of the immune activation phase of the disease. Alternatively, another possible reason could be due to the inhibitory action on the mTOR pathway, which could induce the inhibition of transcriptional processes and consequently induce a reduced viral replication.

By multivariate analysis, it was found that none of the variables considered (diabetes, BMI, duration of immunosuppressive treatment, duration of renal disease, and concomitant heart disease) showed a statistically significant impact regardless of the presence or absence of mTOR inhibitors. Furthermore, as reported in the meta-analysis by Gatti et al. (24) also in our case, there was no increased mortality risk in this category of patients compared to the general population.

We acknowledge that our study presents several limitations, such as the retrospective design, the small sample size, the monocentric cohort, the lack of data on dosages of immunosuppressive therapies, and changes in immunosuppressive therapy during SARS-CoV-2 infection.

## Conclusion

Our real-life study showed a positive impact of therapy with mTOR inhibitors in SARS-CoV-2 infection occurring in patients who underwent kidney transplant. Due to potential antiviral or immunomodulant properties, this class of drugs might be considered a possible weapon in the fight against COVID-19, both in transplant and non-transplant settings. These hypotheses need to be explored in randomized controlled trials.

## Federico II COVID-Team

Ametrano Luigi, Amicone Maria, Borrelli Francesco, Buonomo Antonio Riccardo, Cattaneo Letizia, Conte Maria Carmela Domenica, Cotugno Mariarosaria, Di Filippo Giovanni, Di Filippo Isabella, Esposito Nunzia, Festa Lidia, Fusco Ludovica, Foggia Maria, Gallicchio Antonella, Gentile Ivan, Giaccone Agnese, Iuliano Antonio, Lanzardo Amedeo, Licciardi Federica, Mercinelli Simona, Minervini Fulvio, Nobile Mariano, Piccione Amerigo, Pinchera Biagio, Reynaud Laura, Salemi Fabrizio, Sardanelli Alessia, Sarno Marina, Schiano Moriello Nicola, Scordino Fabrizio, Scotto Riccardo, Stagnaro Francesca, Tosone Grazia, Vecchietti Ilaria, Viceconte Giulio, Zappulo Emanuela, and Zotta Irene.

## Data Availability Statement

The raw data supporting the conclusions of this article will be made available by the authors, without undue reservation.

## Ethics Statement

The studies involving human participants were reviewed and approved by Ethics Committee Federico II. Written informed consent for participation was not required for this study in accordance with the National Legislation and the Institutional Requirements.

## Author Contributions

BP: conceptualization, investigation, writing—original draft, writing—review and editing, and project administration. IG: writing—original draft, writing—review and editing, and supervision. RV: resources, data curation, and validation. LN: data curation, software, and project administration. FC: validation, investigation, and writing—review and editing. RL: formal analysis, data curation, and resources. FP: data curation, software, and resources. ES: formal analysis, data curation, and project administration. FS: software, data curation, and investigation. RC: methodology, resources, and supervision. MF: validation, resources, and project administration. AB: methodology, writing—review and editing, and visualization. LS: methodology, formal analysis, and data curation. All authors contributed to the article and approved the submitted version.

## Conflict of Interest

The authors declare that the research was conducted in the absence of any commercial or financial relationships that could be construed as a potential conflict of interest.

## Publisher's Note

All claims expressed in this article are solely those of the authors and do not necessarily represent those of their affiliated organizations, or those of the publisher, the editors and the reviewers. Any product that may be evaluated in this article, or claim that may be made by its manufacturer, is not guaranteed or endorsed by the publisher.
